# Gut microbiome and its role in colorectal cancer

**DOI:** 10.1186/s12885-021-09054-2

**Published:** 2021-12-11

**Authors:** Martina Rebersek

**Affiliations:** 1 Department of Medical Oncology, Institute of Oncology Ljubljana, Zaloska 2, SI-1000 Ljubljana, Slovenia; 2 Faculty of Medicine, University of Ljubljana, Ljubljana, Slovenia

**Keywords:** Gut microbiome, Colorectal cancer, Prognostic and predictive biomarkers, Modulation of gut microbiome, Systemic treatment of CRC

## Abstract

Colorectal cancer (CRC) is still one of the most common types of cancer in the world, and the gut microbiome plays an important role in its development. The microbiome is involved in the carcinogenesis, formation and progression of CRC as well as its response to different systemic therapies. The composition of bacterial strains and the influence of geography, race, sex, and diet on the composition of the microbiome serve as important information for screening, early detection and prediction of the treatment outcome of CRC.

Microbiome modulation is one of the most prospective new strategies in medicine to improve the health of individuals. Therefore, future research and clinical trials on the gut microbiome in oncology as well as in the treatment of CRC patients are warranted to determine the efficacy of systemic treatments for CRC, minimize adverse effects and increase survival rates.

## Background

Colorectal cancer (CRC) is the third most common type of cancer with almost 2 million new cases per year, and it is the second leading cause of cancer-related deaths worldwide [1]. CRC is also one of the most common cancer types in Slovenia [2]. According to the Cancer Registry of Slovenia, there were 1321 new cases of CRC in 2017, of which 790 cases were men and 531 cases were women [2]. In Slovenia, the incidence of CRC has been declining in the last few years, mostly due to secondary preventive screening programs. Managing a patient with CRC, especially one with a metastatic disease, is complex and expensive, and it can result in a poor quality of life [3, 4]. Thus, primary prevention and screening programmes for CRC are crucial for contributing to a healthy society and for saving lives.

Only 10 to 15% of CRC cases are hereditary, which underlines an important role of the environment as a factor that genetically and epigenetically influences the development of CRC. In recent years, increasing importance in the development of CRC has also been attributed to the gut microbiome.

### Novel classification of CRC and its connection with gut microbiota

**Fig. 1:**
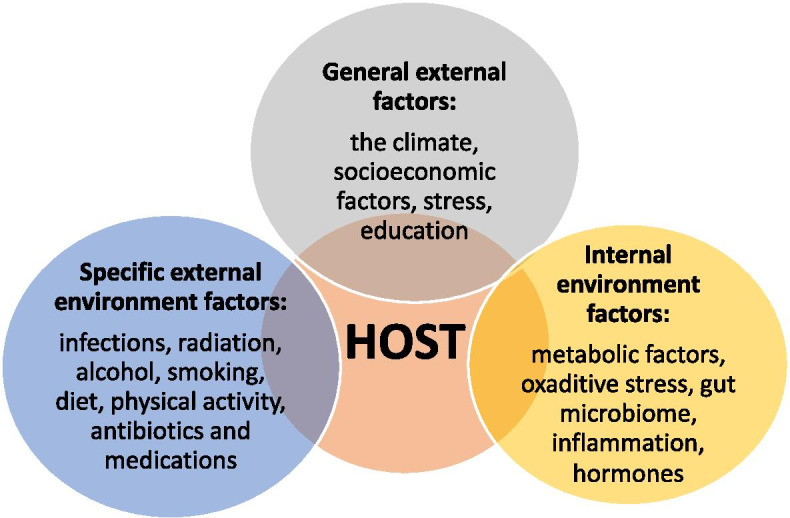
Factors involved in the development of EOCRC are specific external environmental factors, general external factors and internal environmental factors

In recent years, the rise of CRC in those under 50 years of age, known as early-onset CRC (EOCRC), has become an increasing problem. EOCRC is epidemiologically, pathologically, anatomically, metabolically and biologically different from late-onset CRC (LOCRC). The incidence of EOCRC is estimated to increase by more than 140% by 2030 [5–9]. Anatomically, EOCRC is more frequent in the left colon and rectum than LOCRC. Family and hereditary conditions are a factor in 30% of EOCRC compared to approximately 15% in LOCRC. EOCRC also has a different signature than LOCRC as follows: approximately 60% of cases are microsatellite and chromosome stable: higher percentage of *KRAS* and tumour protein p53 (*TP53*) mutations; LINE-1 hypomethylation; and a lower percentage of *BRAF* and adenomatous polyposis coli (*APC*) mutations [5]. There is also a metabolic difference between EOCRC and LOCRC. Increased EOCRC incidence can be the consequence of the generational shift towards a higher body mass index and obesity caused by exposure to carcinogenic factors early in life, such as an interaction of the gut microbiome and inflammation, as well as other specific external factors, such as low-quality food and additive-laden food [5]. Obesity in early life, especially in connection with maternal obesity or obesity during infancy or childhood, can cause dysbiosis and inflammation, thereby leading to EOCRC [5]. Various external and internal factors are involved in this specific EOCRC [5] (Fig. 1).

**Fig. 2:**
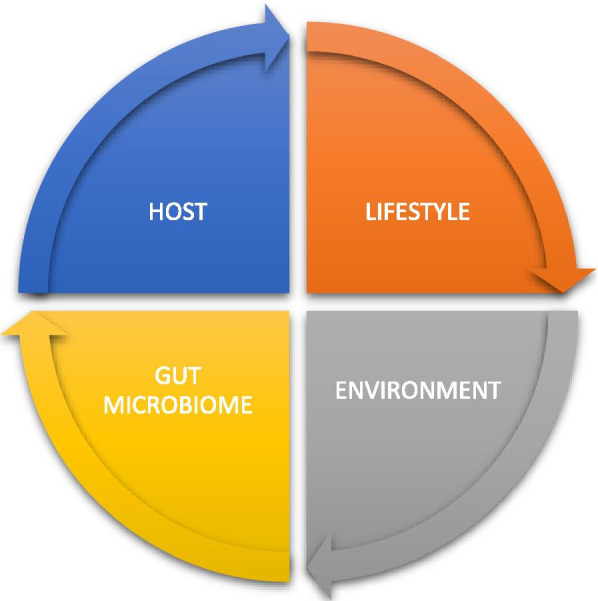
Four main factors involved in dysbiosis of the gut microbiome and the development of CRC are the host and the host’s lifestyle, environment and gut microbiome

General external factors are climate conditions, socioeconomic factors, education and stress. Specific external environmental factors are infections, radiation, alcohol, smoking, diet, physical activity, antibiotics and medications. Internal environmental factors are metabolic factors, the gut microbiome, oxidative stress, inflammation and hormones [5, 9–12]. One of the most important internal environmental factors is the gut microbiome. Under certain circumstances, exposure to an external environmental factor, such as stress or antibiotics, and synthetic food dyes or an internal factor, such as inflammation, leads to dysbiosis in the gut microbiome and consequently to CRC. For example, certain microbiota mediate the effects of a certain diet on CRC risk by generating butyrate, folate and biotin, which play a key role in the regulation of epithelial proliferation. CRC-associated microbiota also contributes to oncogenic epigenetic signatures [5, 13]. Stress, defined as an individual perception of psychosocial stress, is the most important general external factor contributing to the development of EOCRC, causing genetic, epigenetic and microbial changes in the individual as well as in the offspring of a stressed individual. Because psychosocial stress modulates microbiota signatures in gastrointestinal tumours (GITs), stress-induced dysbiosis and inflammatory load lead to the development of EOCRC [6]. Four main factors involved in dysbiosis of the microbiome and consequent development of CRC are the host and the host’s lifestyle, environment and gut microbiome (Fig. 2). One of the important issues in EOCRC is racial disparity. African Americans have a 20% higher incidence of CRC than Caucasians [13], and they are more likely to develop CRC at younger ages. The factors that have been linked with EOCRC, including obesity, physical inactivity, low socioeconomic status and unhealthy dietary patterns, are more prevalent in African Americans [14–16]. African Americans are also more likely to be diagnosed with CRC that originated in the right colon, and the reason for this racial difference is in the epigenome of the right colon relative to the left colon. Differences in the gut microbiome have been increasingly implicated in the rising incidence of EOCRC and may also contribute to higher CRC incidence in African Americans.

### Gut microbiome effectors

The human microbiota consists of a wide variety of microorganisms, bacteria, viruses, fungi and protozoa [13, 17–20]. The gut microbiome consists of microbial cells and their genetic material. The gastrointestinal tract inhabits a population of 10^13^ to 10^14^ different microorganisms and contains over 3 million genes, which is 150 times more genes than in the genome of a human body. The adult gut microbiome consists of more than 1000 different species and more than 7000 different strains of bacteria [21]. The microbiome is a part of each individual from birth when an infant gut is exposed to a complex microflora, which varies depending on the method of delivery. Because the microflora is important for the normal health of an individual, vaginal delivery is preferable as it exposes the infant’s gut to a complex microflora of a mother, resulting in a maternal signature in the initial microbiome of the infant [13, 20].

The gut microbiome has three main functions as follows: structural, protective and metabolic [13, 20]. The gut microbiome plays an important role in the following processes: nutrient and mineral absorption; the synthesis of different enzymes, vitamins and amino acids; and the production of short-chain fatty acids (SCFAs). The fermentation byproducts of gut microbiota, including acetate, propionate and butyrate, are important for gut health, and they provide energy for epithelial cells, enhance epithelial barrier integrity, provide immunomodulation and protect against pathogens [13].

The metabolic axis between the intestinal microbiota and the host is one of the two most important axes in the body. Through its metabolism, microbiota participates in many digestive processes in the gut lumen, such as fibre digestion and metabolism of bile acids, fats and sugars. Thus, the microbiota contributes to the production of important metabolic products, such as vitamins and neurotransmitters, which are important for the functioning of body tissues and organs [21, 22].

Two other important axes are the neuroendocrine hypothalamus-pituitary-adrenal gland (HPA) axis and the axis between the brain and the intestinal microbiota [20]. The communication between the brain and the intestinal microbiota runs both ways; the brain signals affect the motor, sensory and secretory function of the gut, and the intestinal microbiota sends an appropriate reaction through the intestinal nervous system back into the brain. The HPA axis has an important role in the body’s response to psychophysical stresses, and the vagus nerve and intestinal microbiota play an important role in this response. Therefore, it is important that appropriate microbial colonization of the intestine takes place in the earliest years of life.

The alteration of the microbial community is called dysbiosis. Dysbiosis causes altered metabolism in the intestine, thereby disturbing the functions of the microbiota as well as those organs, including the brain [20]. Thus, changes in behaviour, cognitive functions, emotions and nociception can occur in the case of dysbiosis. Furthermore, stress at the level of the brain can, in turn, cause dysbiosis of the microbiota in the gut [20]. This process is the neurochemical behaviour profile associated with the functioning of the intestinal microbiota.

There is also coordinated action and communication between the gut microbiome and the immune system of the host. The gut microbiota enables the immune system to recognize and attack opportunistic bacteria via specific receptors, such as Toll-like receptors, or their metabolites, such as short-chain fatty acids (SCFAs), which promote immunity with IgA production in plasma cells. IgA antibodies block bacterial adherence to epithelial cells and disable further harmful processes. IgA antibodies also directly affect bacterial virulence [19], which prevents bacterial invasion and infection. This system is important for localized immune responses [13, 17–19].

The development and alteration of the gut microbiome are affected by numerous factors, such as the type of infant delivery, type of infant feeding method, the environment, exposure to stress during the lifetime and the individual’s age, diet, potential use of medications and comorbidities [13, 17–19]. Dysbiosis can result in decreased diversity and numbers of commensal bacteria [13, 17–19], and it is connected to a wide array of chronic diseases, such as cardiovascular, metabolic, neurological, autoimmune and gastrointestinal diseases as well as inflammatory bowel disease, obesity and cancer [13, 17–19].

The gut microbiota is comprised of commensal and pathogenic bacteria residing inside the gastrointestinal tract. The four main groups of bacteria in the gut microbiota are *Firmicutes, Bacteroidetes, Actinobacteria* and *Proteobacteria* [13, 20]. Each part of the colon and rectum is characterized by different strains of bacteria. The gut microbiota involved in the development of CRC has different characteristics compared to a healthy microbiota. The most important strains studied regarding the development of CRC are *Fusobacterium nucleatum*, *Escherichia coli* and *Bacteroides fragilis*. The characteristics of gut microbiomes also vary geographically, but many common strains of bacteria connected to CRC development are found in different populations across the world. Among them, the following seven enriched bacterial strains associated with CRC have been identified: *Bacteroides fragilis;* four oral bacterial strains of *Fusobacterium nucleatum*, *Parvimonas micra*, *Porphyromonas asaccharolytica* and *Prevotella intermedia*; *Alistipes finegoldii*; and *Thermanaerovibrio acidaminovorans* [13].

Because the aforementioned topics have been extensively reviewed in other papers, the focus of the present review is CRC, particularly the influence of microbiota on the development of CRC and the outcome of systemic therapy for CRC as well as potential modes of modulation of the microbiota for better treatment outcomes.

### Role of the gut microbiome in the carcinogenesis of colorectal cancer

In the last few years, the role of the microbiome in the development of CRC has been increasingly emphasized [13, 19–21, 23–26]. It is well known that the gut microbiome has an important role in the carcinogenesis of CRC, causing initial inflammation and modulating different signalling pathways [13, 19–21, 23]. Because bacterial biomarkers have the potential to detect CRC and predict clinical outcome, they have prognostic value [13, 19, 25–28]. During the development of cancer, a complex interaction is established among the gut microbiome, tumour microbiome and immune system [18, 26]. The gut microbiome is in a state of health, known as eubiosis, when the following factors are present: diversity of bacteria in the gut microbiota; a balance between proinflammatory and anti-inflammatory cytokines; a balance between immune cells and IgA secretion; and an intact and healthy mucosal barrier and mucus layer. In dysbiosis, these parameters are not in balance. Furthermore, the tumour microbiome has a negative impact on the gut microbiome, causing poor local and systemic responses of the host immune system as well as limited efficacy of systemic treatment with chemotherapy and immunotherapy [20, 25–29].

### Bacteria associated with CRC

The CRC microbiota has a different composition of strains of bacteria than a healthy gut microbiome, and it includes strains individually linked to CRC, such as *Bacteroides fragilis*, *Streptococcus gallolyticus, Enterococcus faecalis* and *Escherichia coli*, as well as other newly found strains of bacteria connected to CRC, such as *Fusobacterium nucleatum*, *Parvimonas*, *Peptostreptococcus*, *Porphyromonas* and *Prevotella*. Higher quantities of these bacterial strains in faecal and tumour samples from patients with CRC tumour microbiota can serve as CRC biomarkers [13, 20]. The gut microbiota influences colorectal carcinogenesis through a variety of mechanisms as follows: inflammation, regulation of immune response and modified metabolism of dietary components, which can also lead to the production of harmful microbial-derived products, such as metabolites or genotoxins [13, 17, 19, 25–28]. Bacteria can be directly procarcinogenic, known as driver bacteria, or indirectly procarcinogenic, known as passenger bacteria. The latter proliferate as opportunistic microorganisms in the tumour-associated microenvironment [13, 29]. Host–to-microorganism interactions contribute to the activation of procarcinogenic signalling pathways that lead to molecular changes and, consequently, to the progression of CRC. These mechanistic components have the potential to be modulated for therapeutic or prophylactic purposes in the context of CRC [13, 20, 21, 29].

**Fig. 3:**
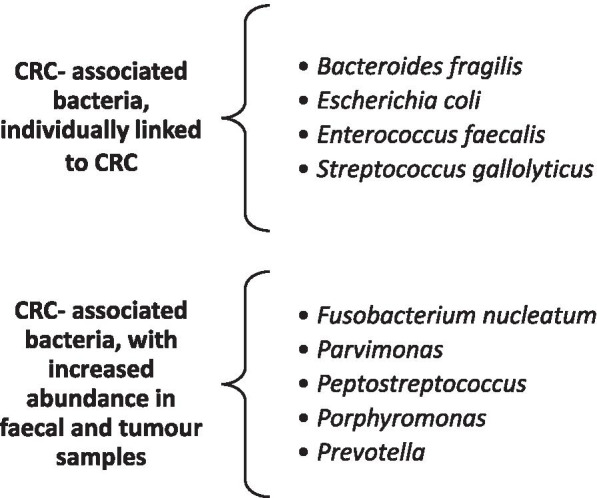
Bacteria linked to CRC are known as CRC-associated bacteria, such as *Bacteroides fragilis*, *Escherichia coli*, *Enterococcus faecalis* and *Streptococcus gallolyticus*, which are individually linked to CRC. Strains of *Fusobacterium nucleatum*, *Parvimonas*, *Peptostreptococcus*, *Porphyromonas* and *Prevotella* have increased abundance in faecal and tumour samples from patients with CRC

Colorectal adenomas, which are precursors of CRC, have also been studied in relation to the gut microbiome [13]. Studies have reported several factors to be specific and important in the development of CRC, namely, the presence of specific bacterial strains, the changes in their composition and the abundance of certain strains, including fungal strains in colorectal adenomas. Some bacterial strains, such as *Fusobacterium nucleatum* and *Solobacterium moorei,* are in high abundance in the early stages of CRC to metastatic disease, and some bacterial strains, such as *Atopobium parvulum* and *Actinomyces odontolyticus,* are in high abundance only in the case of adenomas and intramucosal carcinomas [13]. Studying the microbiome and the biology of colorectal adenomas would help detect, reduce or slow the progression of these diseases to CRC in the future [13]. Furthermore, an epidemiological study by Ahn et al. confirmed the difference in the composition of bacterial strains in the gut microbiota of CRC patients compared to the gut microbiota of healthy persons [30]. Ahn and colleagues examined the extracted deoxyribonucleic acid (DNA) from faecal samples and found that CRC patients have an abundance of *Bacteroides*, *Fusobacterium, Atopobium* and *Porphyromonas* phyla but a depletion of *Firmicutes*. These researchers also pointed out a weakness of the study, namely, that mucosal adherent gut bacteria, which might be more closely linked to colon carcinogenesis than the bacteria in faeces, were not examined [13]. In the future, the results of this study could enable early detection of precursors of CRC and, thus, help prevent its development. The most important strains of bacteria linked to CRC, known as CRC-associated bacteria, are *Bacteroides fragilis*, *Escherichia coli*, *Enterococcus faecalis* and *Streptococcus gallolyticus*, which are individually linked to CRC, and strains of *Fusobacterium nucleatum*, *Parvimonas*, *Peptostreptococcus*, *Porphyromonas* and *Prevotella,* have been identified in increased numbers in faecal and tumour samples from patients with CRC [13, 17] (Fig. 3). Strains of these bacteria have been studied in the past few years with culture-based methods and quantitative real-time polymerase chain reaction (qRT–PCR) using DNA extracted from colorectal tissue biopsies and patient stool samples; more recently, these bacteria have been studied with next-generation sequencing techniques by enabling 16S rRNA gene and metagenomic profiling [13, 17].

Bacteria act procarcinogenic in different ways. *Fusobacterium nucleatum* promotes CRC development through microRNA (miRNA)-mediated activation of Toll-like receptor 2 (TLR2)/Toll-like receptor 4 (TLR4) signalling and the inhibition of apoptosis [31]. *Peptostreptococcus* acts procarcinogenically via its metabolites, which produce more acid and a hypoxic tumour microenvironment as well as enhance bacterial colonization. Some bacteria, such as *Escherichia coli,* are genotoxic, i.e., they damage DNA [13, 17]. Genotoxins, such as cyclomodulin cycle inhibiting factor (CIF), block mitosis and induce apoptosis in epithelial cells. Cytotoxic necrotizing factor 1 (CNF-1) affects the actin cytoskeleton, while colibactin induces DNA double-strand breaks.

Some bacteria are procarcinogenic because they establish interactions between receptors in the host immune system and cancer cells by secreting metabolites, such as secreted proteins called secretomes or metabolites called metabolomes [13, 17]. Secretomes include growth factors, proteases, cytokines and other proteins. Metabolomes include various metabolic products of metabolism of gut microbiota and oncometabolites involved in carcinogenesis. Oncometabolites are metabolic products of microbiota, such as L-2-hydroxyglutarate, succinate, fumarate, D-2-hydroxyglutarate and lactate, and they accumulate in cancer cells after metabolizing. Some metabolites, such as lactic acid, serve as a fuel for cancer cells and cancer progression, while others, such as butyrate, suppress proinflammatory genes and tumour growth [13, 17].

Another important issue is the sidedness of the tumour and bacterial spatialization in CRC. There are differences in the biology, pathology and epidemiology of right-sided (caecum, ascending and transverse colon) and left-sided (descending colon and rectosigmoid junction) CRC, and there are differences in the diversity in the abundance of the microbiome and its potential pathogenic influence on each side of the proximal–distal axis [13, 17]. Right–sided tumours are characterized by the following features: mucinous and signet ring histology; hypermutable microsatellite instable (MSI)-high and CpG island methylator phenotype (CIMP)-high phenotypes; poorly differentiated; infiltrated with immune cells; and have higher mutation rates of phosphatidylinositol-4,5-bisphosphate 3-kinase catalytic subunit alpha (PIK3CA), Kirsten rat sarcoma virus (KRAS) and B-Raf murine sarcoma viral oncogene homologue B (BRAF). These right-sided tumours are more frequent in older and female patients [31–35], and their microbiota invades colonic crypts and consists of the abovementioned CRC-associated bacteria. These CRC microbiome-associated bacteria are also specific for consensus molecular subtype (CMS) 1, which includes all the features listed above. One of four CMS subtypes, including all tumour characteristics, genetics, epigenetics, transcriptomic, clinical features and tumour microenvironment, and in CMS 1, the gut microbiome, as a predictive and prognostic biomarker, can be determined for each individual patient to aid in selecting the best treatment [13, 17, 32–35]. Tumour localization and distribution of bacteria are important for patient prognosis and for the future of planning the treatment of CRC patients as they are involved in the metabolism of chemotherapy and its effectiveness as well as in the immune-related colitis of immunotherapy [32–35].

Not only bacteria but also viruses play a role in carcinogenesis through different mechanisms in different types of cancers, such as lymphomas, Merkel cell carcinoma, cervical cancer and hepatocellular carcinoma. However, it is currently unknown if viruses are involved in the carcinogenesis of colorectal cancer [20, 25].

Diet is one of the most important factors for determining the state of the gut microbiome due to the symbiotic relationship between the gut microbiome and its host in the process of digestion [13, 20]. One of the most important roles of the gut microbiome is food digestion and harvesting the key nutrients that the host is not capable of metabolizing on its own. The microbial metagenome encodes genes that metabolize many nutrients, such as nondigestible carbohydrates, including galacto-oligosaccharides and fructo-oligosaccharides, as well as host-produced compounds, such as bile acids. Studies have reported that 35% of CRC cases are linked to dietary factors, such as poor diet with either low food intake or high intake of refined carbohydrates, added sugars, fats and animal products, especially processed meat.

Different diets modulate the microbiota and consequently affect the intestinal mucosa through the products of nutrient metabolism, which may be protective and anti-inflammatory or proinflammatory, leading to the formation of CRC. The most important nutrient is dietary fibre, which affects gut microbial composition and diversity [13, 20]. Dietary fibre, including fructans and galacto-oligosaccharides, has a tumour-suppressive effect, and it alters gut microbiota composition to increase the abundance of Bifidobacterium and Lactobacillus spp., thereby increasing the faecal butyrate concentration. Thus, dietary fibre acts in a microbiota-dependent and butyrate-dependent manner. In contrast, red meat and processed meat intake are associated with an increased risk of CRC [13, 20]. Red and processed meat contain haem iron, and when haem iron is broken down in the gut, it forms N-nitroso compounds. These compounds damage the cells lining the bowel, which may lead to cancer. In processed meats, nitrates and nitrites, as preservatives, are also broken down into N-nitroso compounds. Dietary fat is another factor that has an impact on gastrointestinal physiology and the composition of gut microbiota [13, 20]. Dietary fat stimulates hepatic secretion of bile acids to facilitate fat emulsification and increase the enterohepatic circulation of bile acids, promoting inflammatory processes and intestinal tumour formation.

Factors, such as insufficient/excessive cooking time, cooking styles (such as frying), excessive cooking temperature and the presence of moisture, are responsible for the generation of proinflammatory and pro-carcinogenic advanced glycation end-products (AGEs), which are highly oxidant compounds, especially those generated from animal-source foods. AGEs are linked to conditions, such as gut dysbiosis, metabolic syndrome, cardiovascular disease, Alzheimer’s disease and EOCRC. Because AGEs are also transferred through maternal blood, their levels in infant blood can rise to levels typical for adults, which, in connection with high oxidative stress and inflammation, can lead to EOCRC [13, 19–21]. The Western diet often causes gut dysbiosis and inflammation [13, 17–19]. Toxic byproducts of microbial metabolism of such a diet, such as *N*-nitroso compounds and hydrogen sulfide, induce epithelial hyperproliferation by disrupting mucus barrier function [13, 19].

Gut commensal bacteria are neither good nor bad per se. It depends on our diet whether our microbiota produces beneficial or deleterious metabolites from digested food. For example, bacteria from *Clostridium* species can produce either butyrate from dietary fibre or bile acids from dietary fat. Depending on the type of food consumed, metabolic byproducts can affect the epithelial barrier or gut integrity, inhibit histone deacetylase, suppress or enhance inflammation, exert tumour suppressive effects or modulate the immune response [13, 20, 21, 25–29]. Compared to processed food, plant-based food and a fibre-rich diet reduce the risk of cancer, cardiovascular diseases and overall mortality [13, 20, 27, 28, 34, 36]. Metabolic byproducts of the gut microbiome derived from such a diet enable numerous tumour- suppressing and immune-modulating effects as follows: good maintenance of the epithelial barrier and gut integrity; induction of T-regulatory cells; inhibition of histone deacetylase; and suppression of inflammation [20].

Diet is also directly linked to obesity, which is a well-established risk factor for CRC. There are many mechanisms involved in obesity that can contribute to the development of CRC, such as insulin or insulin-like growth factor 1 signalling, adipokines, sex hormones and systemic inflammation [13, 20, 25–29]. The gut microbiota has an important role in these mechanisms because it can modify microorganism-derived proinflammatory molecules and oncometabolites. Obesity is also associated with reduced microbial diversity and changes in the composition of gut microbiota [13, 20, 29]. Diet-related obesity causes widespread histone methylation- and acetylation-activating signalling pathways, resembling those in carcinogenesis. Because of these links, weight control in individuals with obesity can profoundly change the gut microbiota and reduce the risk of cancer development [13, 20].

### Microbiota in CRC systemic therapy

#### Systemic chemotherapy

The gut microbiome is increasingly recognized as a predictive factor for the responses to systemic treatment of CRC patients. The gut microbiome is involved in the metabolism of chemotherapy and its pharmacokinetics as well as antitumour activity and regulation of toxicity. The gut microbiome mediates the response to chemotherapy, especially irinotecan, oxaliplatin and 5-flurouracil, which are prescribed as treatments for metastatic CRC [13, 25, 36–39]. The gut microbiome also mediates the immunomodulation response, regulates metabolism, mediates microbial translocation, reduces ecological diversity and establishes resistance to chemotherapy [13, 19, 25, 36–39]. The gut microbiome also plays a part in increasing the toxicity of chemotherapy, for example, by causing irinotecan-induced diarrhoea. SN-38, as an active metabolite of irinotecan, induces an increased abundance of gut bacteria, whose β glucuronidases can convert the SN-38-conjugated inactive form to the active metabolite, which causes diarrhoea [25, 26, 37]. In the human gut, β glucuronidase activity is present, especially in the Firmicutes phylum [35]. By selectively inhibiting this bacterial enzyme, irinotecan-induced diarrhoea could be prevented [13, 25].

Another cytostatic treatment that has been studied is the antimetabolite drug, fluoropyrimidine, which is most often prescribed in CRC [39]. The gut microbiota and its metabolism have an important role in modulating the metabolism of fluoropyrimidine and its pharmacodynamics, depending on the bacterial strains involved. The inhibition of bacterial ribonucleotide metabolism antagonizes drug efficiency, while the inhibition of deoxyribonucleotide metabolism enhances drug efficiency [39]. This effect can also be regulated by dietary nutrients, such as pyrimidines and vitamin B6, and they can alter the efficiency of 5-fluorouracil (5-FU) by disrupting bacterial folate metabolism, impairing 5-FU action and altering folate homeostasis [39].

One of the potential reasons for resistance to standard chemotherapy is cancer stem cells (CSCs), which are a subgroup of cancer cells responsible for chemoresistance and relapse of disease [40, 41]. CSCs are also known as tumour-initiating cells with the ability to self-renew and to differentiate into heterogeneous cancer cell lineages. Chemotherapy induces tumour heterogeneity of both cancer and normal cells inside the tumour. Despite reducing the bulk of cancer cells and inducing apoptosis, a subset of remaining CSCs can survive and differentiate into cancer cells with higher invasiveness, leading to relapse of disease and chemoresistance. CSCs can be recognized by specific markers of normal CSCs for different cancers, including CRC. Different mechanisms responsible for chemo resistance and cancer relapse have been identified, including epithelial mesenchymal transition, hypoxia, tumour environment, and resistance to DNA damage-induced cell death, cancer-associated fibroblasts, inflammation, immune cells, epigenetics, signalling pathways and others. Thus, CSCs are also involved or interfere with gut microbiota metabolism of chemotherapeutic drugs. Intestinal homeostasis of normal intestinal stem cells is influenced by the intestinal microbiota, but the exact mechanisms of interactions between the microbiota and reprogrammed CSCs in the development of CRC are not known [39]. Currently, investigations are focused on the role of specific microbes, which are involved in modification of the microenvironment and CSC transformation in CRC. Novel therapeutic approaches, including microbiota engineering, are under way to target the pathways to differentiate intestinal steam cells. New therapeutic strategies combining therapy targeting CSCs via their specific surface biomarkers and standard chemotherapy in the treatment of cancer patients in clinical trials are warranted.

#### Immunotherapy

The gut microbiome also plays an important role in the efficiency of immunotherapy with checkpoint inhibitors by enhancing the effect of immunotherapy [13, 17, 19, 42–44]. Immunotherapy with checkpoint inhibitors is prescribed only for certain CRC subtypes, namely, for CRC with high microsatellite instability or for DNA mismatch repair-deficient metastatic CRC, which represents approximately 5% of metastatic CRC [45]. However, the gut microbiome can also be associated with the adverse effects of immunotherapy, especially with immune system-related colitis [45, 46]. The effect of the gut microbiome on immunotherapy depends on the strains of bacteria present in the gut. The relationship between the gut microbiome and the response to immunotherapy with checkpoint inhibitors has also been recognized in other types of cancer, such as melanoma, in which immunotherapy with checkpoint inhibitors is well established [42, 43]. Gopalakrishnan et al. examined the oral and gut microbiomes of melanoma patients treated with immunotherapy with checkpoint inhibitors; more specifically, they examined taxonomic profiling, genomic profiling, metabolic function and the gut microenvironment [42]. Gopalakrishnan and colleagues found that responders to anti-programmed cell death protein 1 (PD-1) immunotherapy have a higher abundance of *Faecalibacterium* and *Ruminococcaceae* bacteria than nonresponders, and they reported that responders also have predominating anabolic functions compared to more catabolic functions in nonresponders as well as enhanced local and systemic responses of the host immune system compared to poorer immune responses of nonresponders [43]. As only 5% of CRC patients have microsatellite instability and are, therefore, candidates for immunotherapy with checkpoint inhibitors, we can extrapolate these findings from melanoma patients into the treatment of CRC patients. Prospective clinical trials, including studies on the relationship between the gut microbiome and immunotherapy in CRC patients, are warranted.

Antibiotics are another factor that can have a negative impact on the response of cancer patients, including CRC patients, to immunotherapy with checkpoint inhibitors [47–58]. Because antibiotics are an important and effective treatment for serious infections, they decrease the morbidity and mortality of cancer patients. However, antibiotics are associated with reduced effectiveness of immunotherapy with checkpoint inhibitors, especially in combination with concomitant medications, such as proton pump inhibitors, corticosteroids, and vaccines, which negatively influence the checkpoint inhibitor response [50–58]. There are data on the detrimental effect of broad-spectrum antibiotics on the efficacy of immunotherapy in cancer patients from retrospective studies on lung cancer, kidney cancer and melanoma [20, 50–58]. Pinato et al. performed a small perspective clinical trial and found that prior administration (but not concomitant administration) of antibiotics is connected to decreased treatment responses and overall survival of cancer patients treated with immune checkpoint inhibitors [51]. The data from these small perspective studies can be extrapolated into immunotherapy treatment of microsatellite instability–high CRC patients because there are no data on this association from any known perspective analysis. In a retrospective analysis including different gastrointestinal cancers, such as CRC, Yan et al. found that antibiotics adversely affect the gut microbiome and influence the development and progression of cancer, especially CRC [52]. Different strains of bacteria are connected to different gastrointestinal (GI) tumours; for example, enterotoxigenic *Bacteroides fragilis* is connected to CRC [53, 54]. Previous studies have shown the correlation between the efficiency of immunotherapy and the abundance of these specific strains of bacteria, such as *Bifidobacterium longum* or *Ruminococcaceae bacteria* [52–54]. Thus, it is possible to influence immunotherapy responsiveness by manipulating gut microbiota. Preclinical studies have demonstrated that *Bifidobacterium* promote dendritic cell and CD8+ T lymphocyte infiltration in the tumour microenvironment, thereby promoting the same effects as immunotherapy in terms of elimination of tumours [47]. Because antibiotics can cause dysbiosis of microbiota and consequently inhibit its ability to modulate the host immune system, both locally and systemically, it is crucial to explore the details of the correlation among the gut microbiota, antibiotics and immunotherapy. To date, only data from retrospective analyses of clinical trials treating melanoma, lung and kidney cancer patients with immunotherapy offer details on this relationship, but these analyses did not provide clear answers as to which antibiotics are the key ones, when to prescribe them or for how long [50–58].

Prospective studies are bound to identify the exact mechanism of antibiotic-related effects on the immunotherapy response, which would enable the development of strategies for the safe prescription of antibiotics to immunotherapy-treated cancer patients.

### Potential applications of the gut microbiome in clinical practice

**Fig. 4:**
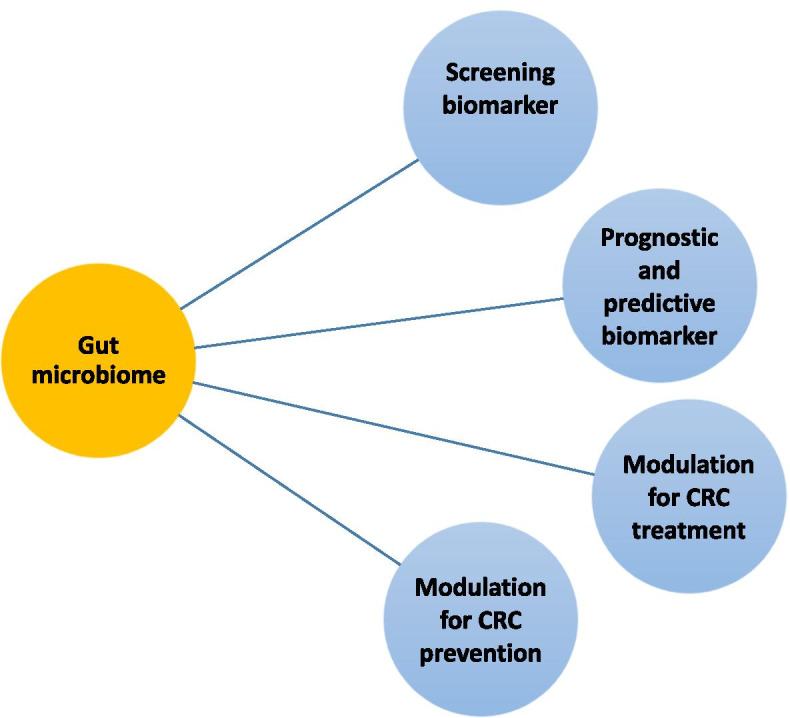
Potential clinical application of the gut microbiota in CRC treatments as a screening, prognostic and predictive biomarker and its possible uses for CRC prevention and CRC treatment

The gut microbiome has many potential roles in dealing with CRC; for example, the gut microbiome may be a screening, prognostic and/or predictive biomarker, or it may be a modifiable factor influencing CRC prevention or CRC systemic treatment effectiveness [13, 25] (Fig. 4).

As a screening marker, the gut microbiota serves as a detector of high-risk adenomas or CRC in asymptomatic individuals [13]. Specific strains of bacteria can serve as screening markers, for example, *Fusobacterium nucleatum*, which can be studied from faecal samples, in which a higher abundance is found in adenomas and CRC patients. Other screening markers, such as metabolic and genotoxic metabolites of specific strains, may serve to recognize and screen CRC in its early stages [13].

As a prognostic and/or predictive biomarker, the gut microbiome may predict the clinical outcome of the patients, their response to the treatment and the possible adverse effects of the treatment [13, 25]. Possible biomarkers may be microbial genes, metabolites and microbiota-related serological markers found in samples of blood, tumour tissue and faeces as well as in samples taken from the oral cavity.

**Fig. 5:**
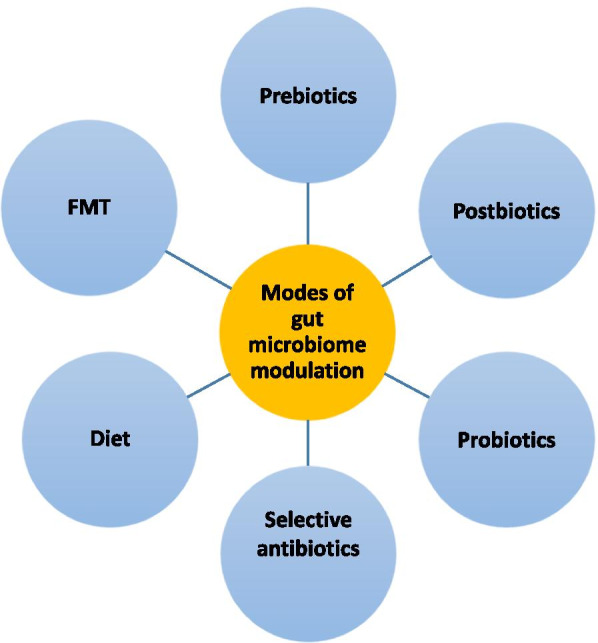
Possible approaches to modulation of the gut microbiome include diet, prebiotics, probiotics, postbiotics, selective antibiotics and FMT

By modulating the gut microbiome, CRC can be prevented in high-risk populations, and responses to chemotherapy and immunotherapy can be improved. In addition, modulation of the gut microbiome can reduce the potential adverse effects of chemotherapy and immunotherapy. Modulation of the gut microbiome can be achieved by dietary intervention, prebiotics, probiotics, postbiotics, antibiotics and faecal microbiota transplantation (FMT) [13, 25] (Fig. 5).

### Modulation of gut microbiota

The gut microbiome can be reshaped by dietary intervention. This includes an intake of prebiotics (such as dietary fibre), a low intake of fat, a plant-based diet, a low or no intake of red and processed meat or a higher intake of probiotics and postbiotics (such as microbial fermentation components, including short-chain fatty acids (SCFAs). These dietary requirements must be combined with weight reduction and exercise [13, 25].

Probiotics are live microorganisms that provide health benefits by improving or restoring the gut flora when administered in adequate amounts [25]. In the case of CRC, preclinical studies have shown several types of bacteria, such as *Bifidobacterium* and *Lactobacillus* spp., to have anticancer functions, including inhibition of cell proliferation, induction of cancer cell apoptosis, modulation of host immunity, deactivation of carcinogenic toxins and production of anticarcinogenic compounds, such as butyrate [13, 25]. Probiotics are widely used in the general population as a food supplement. In 2002, the Food and Agriculture Organization/World Health Organization defined them as live microorganisms that confer a health benefit when consumed in adequate amounts [59].

Probiotics have many functions, such as protecting against pathogenic microbes, maintaining intestinal integrity, participating in intestinal metabolic processes, anti-inflammatory actions, stimulating the immune system response and affecting the signalling pathway between the gut and the central nervous system, thus promoting anxiolytic, antidepressant and nociceptive action [59]. In recent years, probiotics have been prescribed as prevention or treatment for various diseases, such as acute antibiotic-associated diarrhoea, *Clostridium difficile*–associated diarrhoea, autoimmune diseases, cardiovascular diseases and respiratory infections. Probiotics are also used to reduce certain health risks, such as neonatal late-onset sepsis. Nevertheless, preclinical and clinical studies have not confirmed the benefit of probiotics [59]. Many questions on probiotics remain to be answered as follows: which strains of bacteria should be used for treatment; what is the correct ratio of used strains; what kinds of activities individual strains perform; what are the intestinal colonization and the physiological effects associated with probiotics; what interactions they would establish with the intestinal microbiome; and what potential safety issues their usage presents; and how they impact the host [59].

Prebiotics are defined as nondigestible food ingredients that beneficially affect the gut microbiome by selectively stimulating the growth and/or the activity of one or a limited number of bacteria in the colon and, thus, improve host health [60]. In combination with a prebiotic, such as inulin, the *Lactobacillus rhamnosus* GG and *Bifidobacterium lactis* Bb12 probiotics induce changes in the faecal microbiota, increasing the number of beneficial *Lactobacillus* and *Bifidobacterium* strains and decreasing that of the harmful *Clostridium* strain [13, 25, 29, 60]*.*

Postbiotics are microbial fermentation components, including metabolites, short-chain fatty acids (SCFAs), microbial cell fractions, peptidoglycan-derived muropeptides, functional proteins, extracellular polysaccharides (EPS), cell lysates, teichoic acid and pili-type structures [61]. Postbiotics serve as enhancers of the potency of prebiotics, and one potential postbiotic is oncomicrobiotics, the cocktail of bacteria or bacterial products that improves the immune response [31].

Selective antibiotics may also play an important role in the prevention of CRC [13]. By modulating the gut microbiome, antibiotics can act as inhibitors of cancer-associated bacteria, supplement commensals to potentiate cancer therapies or act as small molecule inhibitors to reduce treatment adverse effects. One of the specific and selective treatment possibilities is antibiotic treatment of cancer-associated *F. nucleatum,* in which strains are sensitive to several antibiotics, such as some B-lactam antibiotics, metronidazole and clindamycin, among which metronidazole is most effective in reducing tumour volume in CRC [31, 62].

However, as broad-spectrum antibiotics have a well-known detrimental effect on immunotherapy responses, it is important to combine these selective antibiotics with other gut microbiome modulating modes, such as diet, prebiotics and probiotics or faecal microbiota transplantation, for the best results [13, 30].

Faecal microbiota transplantation (FMT) is the administration of healthy microbiota from a donor into the patients’ intestine as treatment. FMT represents the most direct manipulation of gut microbiota [13, 25, 63–66]. FMT preparations can be administered to patients via oral administration of lyophilized or frozen capsules or via direct infusion of faecal suspension by gastroscopy or colonoscopy. The FMT technique has already been employed as a treatment for patients with *Clostridium difficile* infection (CDI), patients who are resistant to conventional therapies and for patients with chronic inflammatory bowel disease [13, 25, 63–66]. FMT is successful in CDI with a cure rate of more than 90%. Furthermore, the Food and Drug Administration deemed CDI the only approved indication for FMT in the United States in 2013 [66].

FMT is a strictly regulated process that is defined in the international consensus guidelines for FMT, which regulates the entire process as follows: the selection and screening of donors; donor blood and stool testing; the collection, preparation and storage of faeces; and the introduction of FMT into clinical practice [59]. To have faeces available when needed, it is important to establish a stool bank for freezing faeces. FMT is also being tested as a treatment for other diseases with intestinal dysbiosis, predominantly for intestinal diseases but also for metabolic, neurological, cardiovascular and rheumatological diseases [13, 25, 63–66].

Other novel approaches for modulation of the gut microbiome are being introduced as follows: bioengineering the gut microbiota; the synthesis and delivery of genetically engineered probiotics; and presenting bacteriocins or bacteriophages as modifiers of the gut microbiota [13, 25].

## Conclusions and future directions

Predictive and prognostic biomarkers are important in personalized medicine of CRC patients. The gut microbiome is one of them because it can be involved in the carcinogenesis of CRC and can predict the prognosis and response of CRC patients to a specific systemic therapy. There are many interventional approaches to modulating gut microbiota, and many of which have previously been studied in clinical trials, including probiotics, prebiotics, antibiotics, FMT and lifestyle modifications, such as dietary modification and physical activity. Clinical trials have also investigated the modification of microbiota for detecting CRC or adenoma in asymptomatic individuals, improving immunotherapy or chemotherapy responses or reducing their adverse effects. Many questions remain unanswered about the appropriate delivery, kinetics efficiency and durability of modulation of the gut microbiome with prebiotics, probiotics and FMT. The methods of comparing and combining these treatment modes also remain to be studied. In today’s precision medicine, the key step will be the shift from an empirical approach of “one form of probiotic is suitable for everyone” to a personalized approach for each individual.

Furthermore, it is important to emphasize the meaning of primary preventive measures against the development of CRC during infancy and childhood (EOCRC), including a healthy environment, proper diet, exercise, weight control, avoiding stress or relieving stress with relaxation techniques. The implementation of secondary preventive programmes in combination with the aforementioned primary preventive approaches is important for the prevention and early detection of LOCRC.

In the near future, the gut microbiome will have important clinical implications for CRC prevention, planning of systemic treatment and reduction of its adverse effects. The gut microbiome varies geographically, ethnically and according to the dietary habits and lifestyles of individuals. Clinical research will be needed in the near future to include influences on the microbiome of patients, such as geography, race, sex and diet, as well as how it is affected or altered by cancer systemic treatment, especially chemotherapy and immunotherapy. One of the most promising and challenging fields of research is the interaction of gut microbiota and CSCs in the development of CRC. In addition, the implementation of this knowledge and new therapeutic approaches in clinical research and everyday clinical practice will also be challenging. As each individual has a specific gut microbiome since birth, patient-tailored personalized medicine aided by artificial intelligence and machine learning is optimal to ensure better results. It is also important to emphasize that by promoting one’s healthy gut microbiome, the overall health of an individual is improved, which in turn beneficially impacts the public health of the whole society.

## Data Availability

Not applicable.

## Abbreviations

CRC: Colorectal cancer
SCFAs: Short-chain fatty acids
HPA: Hypothalamus-pituitary-adrenal gland
DNA: Deoxyribonucleic acid
qRT–PCR: Quantitative real-time polymerase chain reaction
MSI: Microsatellite instable
CIMP: CpG island methylator phenotype
PIK3CA: Phosphatidylinositol-4,5-bisphosphate 3-kinase catalytic subunit alpha
KRAS: Kirsten rat sarcoma virus
BRAF: B-raf murine sarcoma viral oncogene homologue B
miRNA: microRNA
TLR2: Toll-like receptor 2 (TLR2)
TLR4: Toll-like receptor 4 (TLR4)
CIF: Cyclomodulin cycle inhibiting factor
CNF-1: Cytotoxic necrotizing factor 1
CMS: Consensus molecular subtype
AGEs: Advanced glycation end-products
EOCRC: Early-onset colorectal cancer
LOCRC: Late-onset colorectal cancer
TP53: Tumour protein p53
APC: Adenomatous polyposis coli
GIT: Gastrointestinal tract
CSCs: Cancer stem cells
5-FU: 5-fluorouracil
PD-1: Programmed cell death protein 1
FMT: Faecal microbiota transplantation
GI: Gastrointestinal
EPS: Extracellular polysaccharides
